# Rising trends in the use of frozen dog sperm: a retrospective study in Belgium and the Netherlands

**DOI:** 10.3389/fvets.2024.1499266

**Published:** 2024-11-13

**Authors:** Guillaume Domain, Maarten Kappen, Amber Van Mil, Ilse De Beijer, Matthieu Van Puyvelde, Robby Van Leeuwenberg, Lotte Spanoghe, Florin Posastiuc, Ann Van Soom

**Affiliations:** ^1^Department of Internal Medicine, Reproduction and Population Medicine, Faculty of Veterinary Medicine, Ghent University, Merelbeke, Belgium; ^2^Cryolab Eersel B.V., Eersel, Netherlands

**Keywords:** sperm, cryopreservation, dog, storage, sperm bank

## Abstract

**Introduction:**

Sperm cryopreservation is a valuable technique for storing valuable canine genetics. However, little is known concerning the fate of frozen sperm stored in a sperm bank. This study aimed to characterize dogs presented for sperm cryopreservation and describe the use and popularity of frozen sperm in the Netherlands and Belgium over recent years.

**Methods:**

Medical records from dogs presented for sperm cryopreservation between January 1, 2014 and December 31, 2022 at two different freezing centers were reviewed retrospectively. Imported frozen sperm was excluded due to lack of usage information. Each sperm cryopreservation was considered a single event, and data were collected separately for each cryopreserved sample.

**Results:**

A total of 3,090 ejaculates from 1,040 males of 157 different breeds were included and investigated using exploratory data analysis. The findings showed a steady rise in the popularity of sperm cryopreservation, with annual growth rates ranging from 8.4 to 41.9%. The majority of dogs (88.5%) were between 1 and 9 years old at the time of sperm cryopreservation, with nearly one-third aged 2–4 years. Most dogs were collected for sperm cryopreservation once (62.8%) or twice (21.6%). Sperm banks were used for both short- and long-term storage needs, and 6.83% of ejaculates were eventually discarded. The primary use of froze sperm was for international shipment, while 21.8% was used locally for artificial insemination. Depending on the year of cryopreservation, between 44.1 and 79.6% of frozen ejaculates remained unused or only partially used at the time of data collection.

**Discussion:**

The results of this study provides the first comprehensive analysis of the use and popularity of frozen sperm among dog breeders in Belgium and the Netherlands and suggest a change in breeding practices in recent years. The percentage of breeders resorting to sperm cryopreservation and the extent of frozen sperm use in current breeding strategies remain to be defined in future studies.

## 1 Introduction

Sperm cryopreservation offers the opportunity to preserve the gametes of dogs with high genetic or sentimental value and to overcome the temporal and geographical limits of traditional dog breeding (1–3). With advancements in insemination techniques and satisfactory pregnancy rates—approaching 80% following insemination with frozen-thawed sperm (4)—sperm cryopreservation has become increasingly appealing to breeders, providing access to international markets and facilitating strategic breeding decisions, particularly in managing valuable bloodlines (5, 6).

Frozen sperm offers greater flexibility in breeding, allowing its use even after the dog has experienced age-related decline of fertility, sterility—whether pathological or elective—or even death (1, 7). It also enables the preservation of genetics from disease-free donors with valuable traits, making it possible to establish open access to these genetics when the owner does not wish to breed their dog (8). Transporting frozen sperm instead of live animals reduces stress, minimizes sanitary restrictions, and circumvents potential quarantine requirements associated with live animals transport (2, 7). Additionally, cryopreservation maximizes the return on an ejaculate, as it may be divided into insemination doses and used for different inseminations, sometimes several years apart. However, the efficiency of sperm cryopreservation depends on the quantity and quality of sperm before and after cryopreservation, which can vary significantly between dogs and individual ejaculates (9, 10).

The storage of frozen sperm in liquid nitrogen tanks has led to the development of dedicated facilities known as sperm banks. Initially established at universities, sperm banks have expanded to private companies that offer breeding services and manage the daily international trade of frozen sperm (11). Today, they play a crucial role in enhancing genetic diversity in dogs, particularly important among rare breeds and working dogs that are represented by a limited number of individuals, often geographically distant from one another (12). Dog breeders utilize sperm banks for both personal and commercial purposes, selling sperm to other breeders as opportunities arise. Recently, several assistance dog organizations have integrated sperm cryopreservation into their breeding strategies, freezing sperm from young male dogs before castration to preserve valuable genetics. Similarly, pet owners may seek sperm freezing services for their aging dogs when they wish to preserve the option of having a future litter but lack an immediate breeding opportunity, though sperm quality in elderly dogs is often insufficient for freezing (9, 10, 13).

While several studies have documented the satisfactory fertility results following artificial insemination with frozen-thawed sperm, the specific use and management of frozen dog sperm stored in a sperm bank remain to be described (5, 6, 14–16). This study aimed to characterize dogs presented for sperm cryopreservation and describe the use and popularity of frozen sperm in the Netherlands and Belgium over recent years.

## 2 Materials and methods

### 2.1 Patients and inclusion criteria

Medical records from two sperm freezing centers, Cryolab Eersel in the Netherlands and Ghent University in Belgium, were retrospectively reviewed in July 2023 to identify sperm freezing procedures conducted between January 1, 2014, and December 31, 2022. The start date was decided upon the availability of computerized medical records for all cryopreservation procedures, while the end date ensured adequate follow-up time for the use of frozen sperm. Exclusion criteria included imported frozen sperm due to incomplete data on the utilization of the entire ejaculate. Each sperm cryopreservation event was considered distinct, and data were recorded separately. In total, 3,090 ejaculates (2,957 from Cryolab and 133 from Ghent University) from 1,440 males of 157 different breeds recognized—either definitively or provisionally—by the Fédération Cynologique Internationale were included in the study. The most representative breeds were Belgian Shepherd Dogs—Malinois (*n* = 215), Golden Retriever (*n* = 59), Labrador Retriever (*n* = 58), German Shepherd (*n* = 51), Dutch Shepherd (*n* = 42), Border Collie (*n* = 41), Bullmastiff (*n* = 39), Belgian Shepherd Dogs—Tervueren (*n* = 38), French Bulldog (*n* = 33), and Rottweiler (*n* = 31). Detailed information on the number of dogs for each breed can be found in Supplementary Table 1.

### 2.2 Data collection

Data collected for each cryopreservation procedure included: the date of sperm collection, the birth date of the dog, breed, the number of straws frozen, detailed utilization of the frozen straws, and the number of remaining straws. From these data, additional data were calculated: the age of the dog at sperm collection, the number of freezing procedures per individual, the storage period before the first use, and the storage period before the last use if no straws remained.

If straws were discarded, the disposal date, number of discarded straws, and the reason for disposal were recorded. Subsequently, the storage period before disposal was calculated.

### 2.3 Statistical analysis

Data from the computerized record systems of both freezing centers were exported to a Microsoft Excel spreadsheet (Microsoft Corporation, Redmond, USA). Missing data ranged between 0 and 2.1% depending on the variable, and ejaculates with missing data were excluded from the analysis of the corresponding variable. Exploratory data analyses were performed using the Base and rstatix packages for descriptive statistics, while visualizations (histograms, box plots, and Q-Q plots) were created using ggplot2 (17). Results are presented as median and interquartile range (IQR). To examine trends in the number of cryopreserved ejaculates over time, a linear regression analysis was performed, with statistical significance set at *P* < 0.05.

## 3 Results

### 3.1 Popularity of sperm cryopreservation

**Figure 1:**
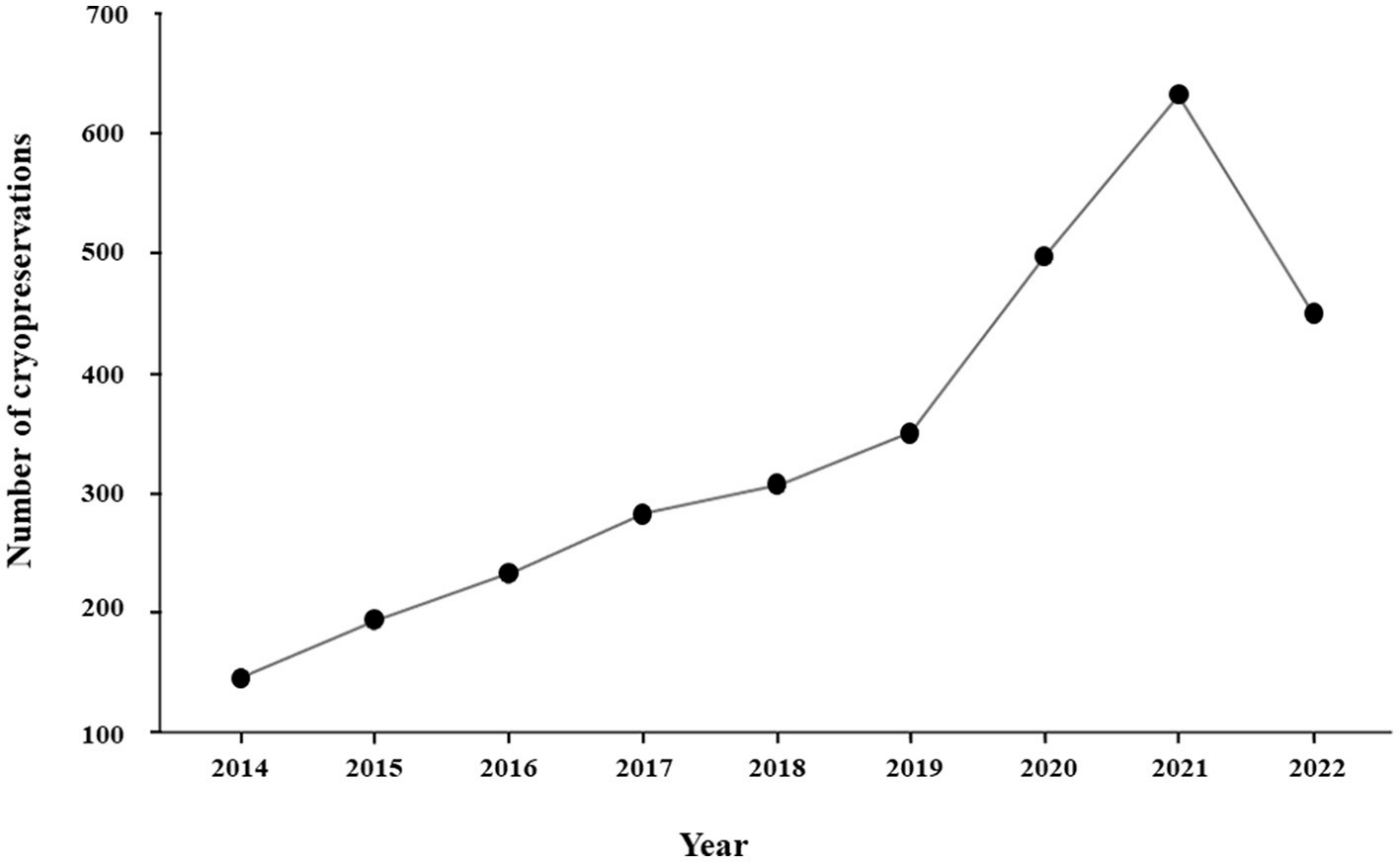
Evolution of the number of cryopreservations between 2014 and 2022.

Since 2014, the number of cryopreservations has increased steadily each year, with annual growth rates ranging from 8.5 to 41.9%. By 2019, the number of ejaculates cryopreserved was 2.4 times higher than in 2014. A sharp rise occurred between 2019 and 2021, nearly doubling (1.8 × ) within 2 years. However, in 2022, the number of freezing procedures decreased by 28.8% compared to 2021 (Figure 1; Supplementary Table 2). Despite the decline in 2022, linear regression analysis over the 9-year study confirmed a significant positive trend in cryopreserved ejaculates (*P* < 0.001), with an average annual increase of 52.32 ejaculates.

### 3.2 Age at collection

**Figure 2:**
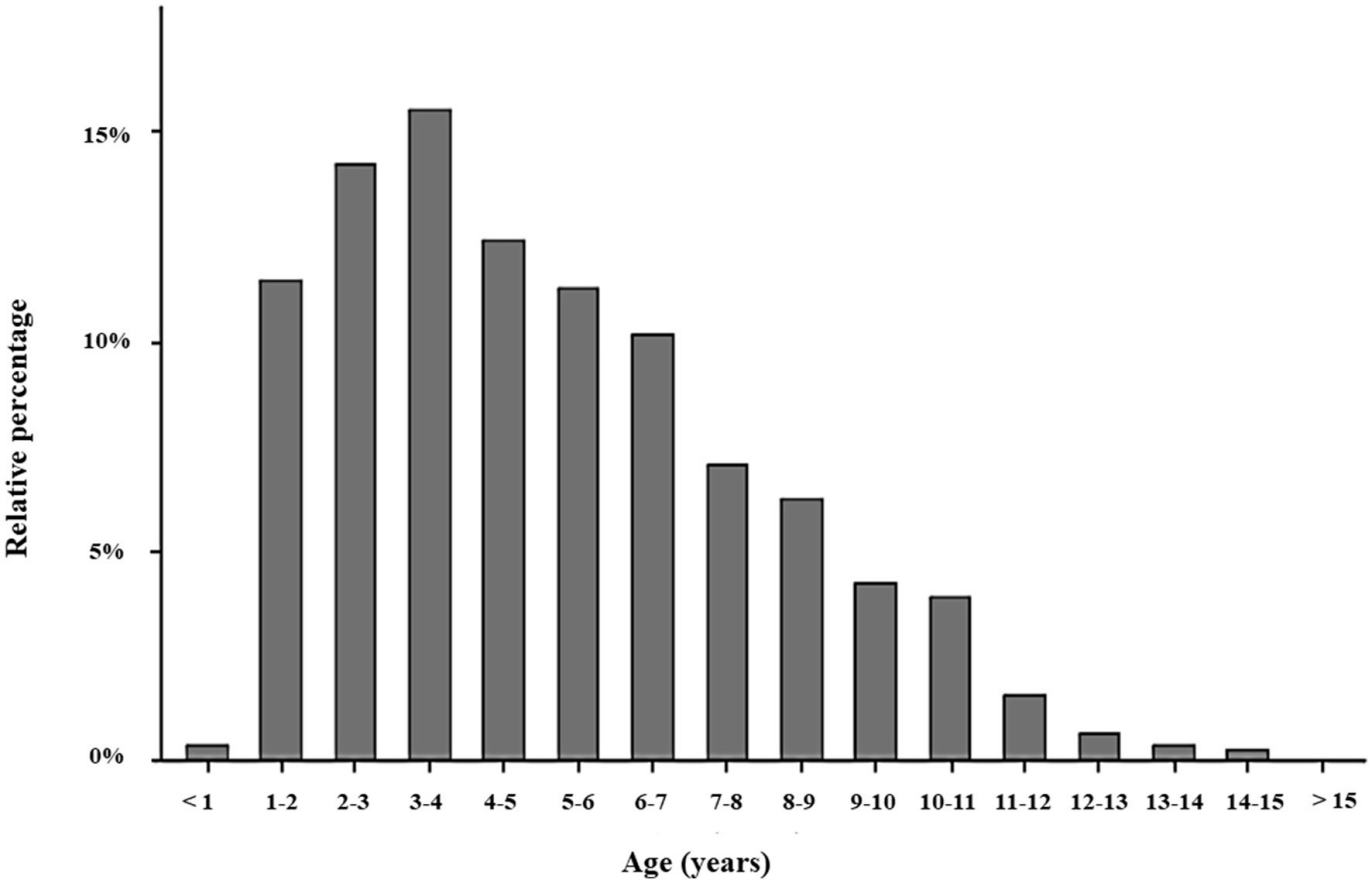
Bar plot representing the proportion of dogs, in %, for each age range at the time of sperm cryopreservation.

Dogs were collected for sperm cryopreservation at a median age of 55 months (IQR: 34–83 months), with ages ranging from 8 to 181 months. The majority of dogs (88.5%) were collected between 1 and 9 years of age, with a notable peak (15.6%) observed between 3 and 4 years. Nearly one-third of the dogs were collected between 2 and 4 years of age. A small proportion of dogs were collected before 1 year of age (0.4%) or after 11 years of age (2.9%) (Figure 2; Table 1).

**Table 1:** Frequency, relative percentage, and cumulative relative percentage of dogs for each age range at the time of sperm cryopreservation.

**Age at collection (years)**	**Number of dogs**	**Relative percentage (%)**	**Cumulative relative percentage (%)**
< 1	12	0.40	0.40
1–2	347	11.46	11.86
2–3	431	14.24	26.10
3–4	471	15.56	41.66
4–5	376	12.42	54.08
5–6	342	11.30	65.38
6–7	308	10.18	75.55
7–8	214	7.07	82.62
8–9	190	6.28	88.90
9–10	129	4.26	93.16
10–11	119	3.93	97.09
11–12	48	1.59	98.68
12–13	20	0.66	99.34
13–14	11	0.36	99.70
14–15	8	0.26	99.97
15–16	1	0.03	100.00

### 3.3 Frequency of cryopreservation

The median number of cryopreservations per dog was 1 (IQR:1–2), ranging from 1 to 66. Most dogs had their sperm cryopreserved once (62.8%) or twice (21.6%), while only a small percentage (1.9%) had their sperm cryopreserved more than six times.

### 3.4 Use of frozen sperm

At the time of data analysis, 1,074 of the 3,090 frozen ejaculates (34.8%) were fully used, 626 (20.3%) were partially used, and 1,390 (45.0%) remained unused in the sperm banks. A total of 2,197 unique uses were recorded, comprising 1,605 international shipments (73.0%), 480 artificial inseminations (21.8%), and 112 national ownership transfers (5.1%).

**Figure 3:**
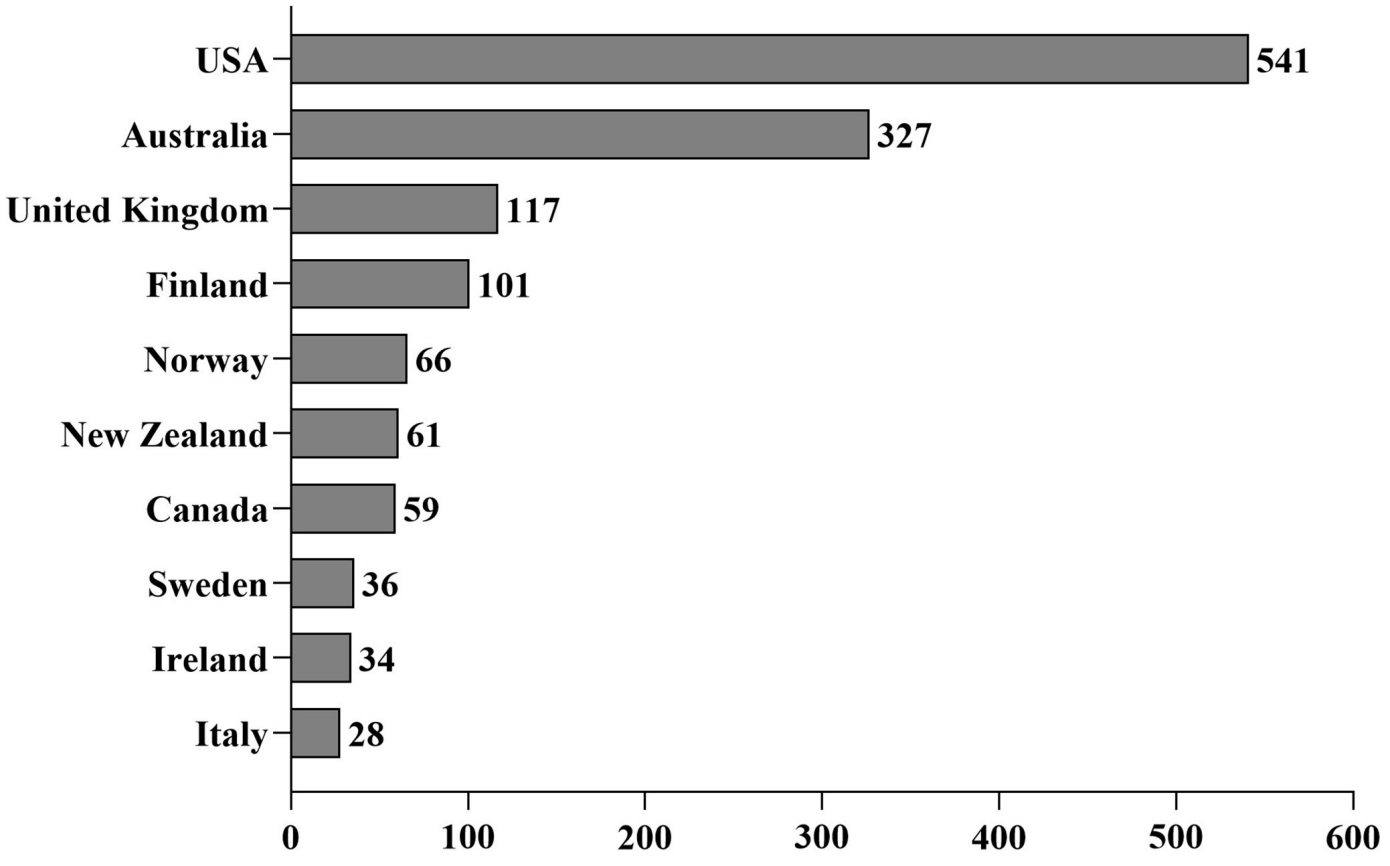
The number of international shipments of frozen sperm to the ten most popular destinations.

When frozen ejaculates were exported, 59.7% were shipped as whole ejaculates (all the straws obtained from the ejaculate) after a median storage time of 60 days (IQR: 12–168) in the sperm bank. The most frequent destinations for international shipments were countries outside the European Union (EU) (Figure 3). When ejaculates were used at the freezing center, artificial inseminations were performed after a median storage period of 342 days (IQR: 114–1,084).

Over the 9-year study period, 211 ejaculates (6.8%) were discarded, primarily due to poor sperm quality after thawing. Other reasons included increased storage fee, identification of a (presumed) inherited condition in the male dog or previous litters, lack of need following a successful pregnancy or natural breeding availability, shifts in breeding strategies involving frozen sperm, or owner dissatisfaction with fertility results after artificial insemination. Discards occurred after a median storage time of 803 days (IQR: 403.5–1,273.5), mostly as whole ejaculates (69.2%).

**Figure 4:**
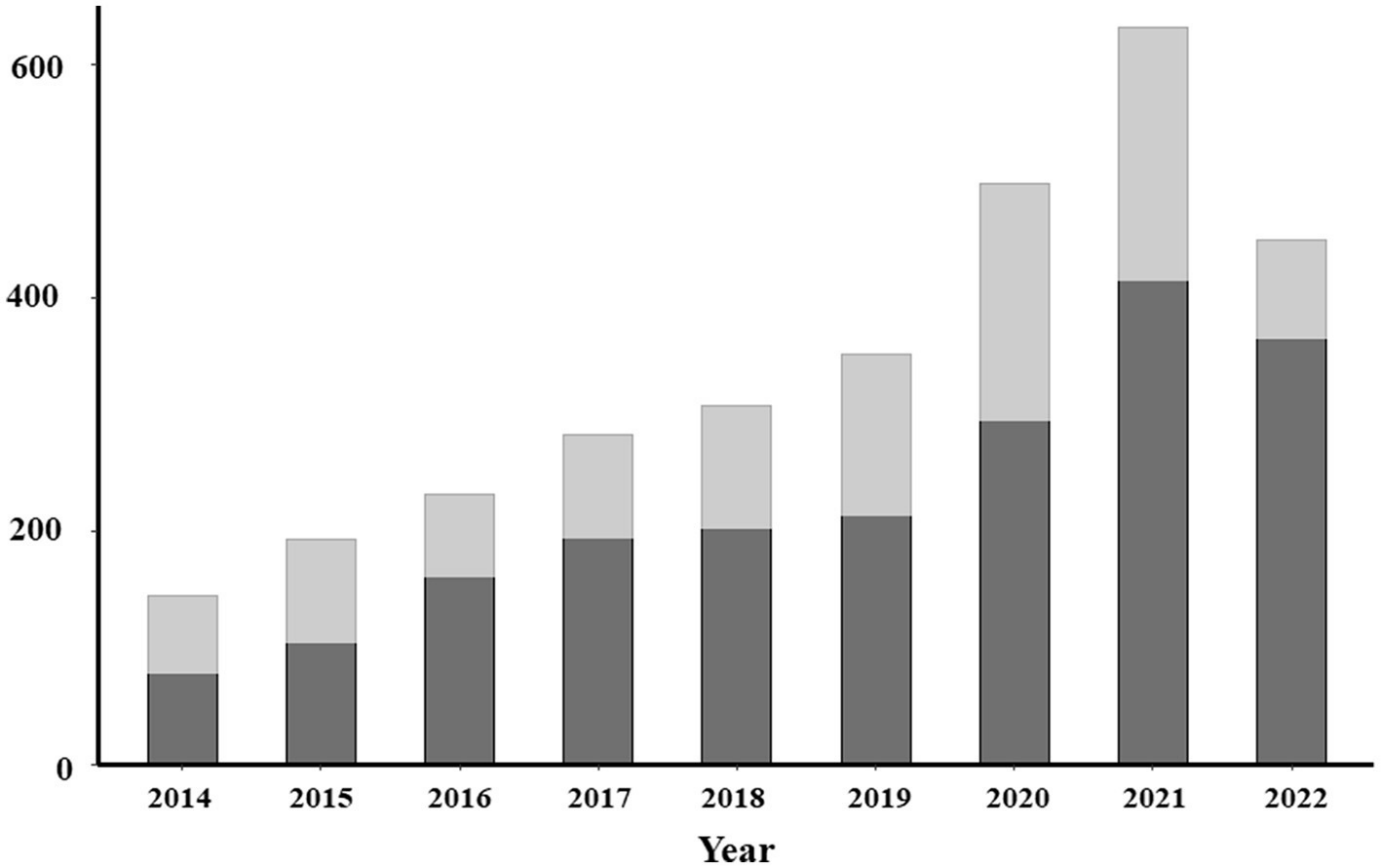
Bar plot representing the number of cryopreservations between 2014 and 2022. The dark gray represents the proportion of remaining frozen ejaculates from each year in the sperm banks.

The relative percentage of remaining ejaculates varied between 44.1 and 79.6% depending on the year of cryopreservation, with lower percentages observed for longer storage durations (Figure 4). Detailed year-by-year percentages of remaining frozen ejaculates are provided in Supplementary Table 2.

## 4 Discussion

The present study characterizes the population of dogs presented for sperm cryopreservation and highlights the growing popularity of this reproductive technology among breeders in the Netherlands and Belgium over the past 9 years. The consistent annual increase in sperm cryopreservation suggests a rising interest among dog breeders in enhancing their breeding program by incorporating genetic material from diverse and geographically distant bloodlines. This trend aligns with previous findings that reported increased gene flow between countries based on pedigree database analyses (18). However, it is important to consider that a potential increasing reputation of the freezing centers examined in this study may have also contributed to this upward trend.

The significant difference in the number of ejaculates collected by Cryolab (2,957) and Ghent University (133) could be attributed to several factors, including Belgium's smaller demographic size and the presence of multiple freezing centers, which likely diffuse the caseload. In contrast, Cryolab is the only dedicated freezing center in the Netherlands and a private veterinary practice with a strong focus on sperm freezing and international export, which likely attracts a higher volume of clients.

The breeds most represented in this study—Belgian Shepherd Dogs, Dutch Shepherds, and German Shepherds—are primarily working dog breeds known for their roles in police, military, and security services. Popular pet breeds, such as Golden Retrievers and Labrador Retrievers, were also well-represented. This breed distribution suggests that cryopreservation is gaining traction not only among working dog breeders but also among those of popular family pets.

The significant spike in sperm cryopreservations observed in 2020 and 2021 was likely influenced by the global COVID-19 pandemic, which caused a marked increase in the demand for puppies (19). At the same time, restrictions on physical contact and border closures between countries limited direct interactions between breeders. Consequently, sperm cryopreservation and international shipments became an effective alternative to maintain breeding schedules and genetic exchange during this period. Although cryopreservation numbers declined in 2022, this decrease does not overshadow the overall trend of steady growth, supported by an average annual increase of 52.32 cryopreserved ejaculates. The drop in 2022 likely reflects a return to pre-pandemic norms rather than a reversal in the growing use of frozen sperm.

Most sperm cryopreservation were from dogs aged 1–9 years, with nearly a third (29.8%) between 2 and 4 years old. This age distribution likely reflects the inclusion of young, recently certified breeding males that have proven their worth in shows or working competitions (20). In contrast, fewer than 10% of the dogs were 10 years or older, aligning with evidence that sperm quality generally declines with age (10, 13, 21–24). However, the age distribution may be skewed toward younger dogs, as it excluded those initially presented for cryopreservation but deemed unsuitable due to poor semen quality. In some cases, some owners may choose to freeze sperm from older dogs despite reduced semen quality, driven by emotional attachment or unique breed traits. Nonetheless, achieving pregnancy in such cases is unlikely without advanced reproductive technologies, like *in vitro* fertilization, and the advanced age of the dog may further impair sperm fertility (24–26). Additionally, surgical artificial insemination is illegal in several European countries, including the United Kingdom, Norway, Sweden, and the Netherlands (27).

Most dogs were presented only once or twice for sperm cryopreservation. However, the case of one dog presented 66 times highlights the intense use of certain sires by some breeders. Such practices are not recommended, as they pose significant risks to genetic diversity and can facilitate the spread of inherited disorders within a breed (12, 28). Fortunately, these extreme cases were rare, with only 1.9% of dogs being presented more than six times. To mitigate these issues, some national breeding organizations have implemented restrictions on the use of specific sires or the total number of breedings allowed per male (29).

Frozen sperm was primarily used for international shipments, after a median storage time of 60 days. This relatively short storage duration indicates that many breeders use sperm banks as temporary storage facilities, awaiting the accumulation of sufficient straws or the finalization of logistical arrangements for shipment. This finding supports our assumption that a 6-month period between the last cryopreservation and data collection was adequate for assessing the use of frozen sperm by dog breeders. This timeframe provided ample time for sperm utilization, while minimizing the risk of missing late usage cases. However, it remains unknown whether the exported frozen sperm is used shortly after arrival or stored for a longer period in another sperm bank. In most cases (59.7%), all straws from a single ejaculate were shipped, which contrasts with practices in other species, such as stallions and bulls, where dividing ejaculates into multiple insemination doses is common practice (30, 31). This difference likely arises from the lower total sperm count in dogs and the relatively high recommended sperm concentration per insemination dose, i.e., 100 million progressively motile and normal spermatozoa (5, 16).

The most popular destinations for international shipment were typically countries with extended transit times (16, 32). International shipment to EU member countries, such as Finland, Sweden, Ireland, and Italy, may reflect a growing popularity of frozen dog sperm in these regions or logistical choices favoring frozen transport due to geographical distances from the investigated sperm banks. In addition to international shipments, artificial inseminations performed at the freezing centers accounted for 21.8% of frozen sperm use. Many owners of frozen sperm likely prefer insemination at the sperm bank to avoid the additional costs and risks associated with shipping, although this option is limited by the location of the bitch. Lastly, 6.8% of the frozen ejaculates were discarded, indicating that a small but notable proportion of breeders ultimately decided to discontinue sperm storage for various reasons.

A significant portion of frozen ejaculates (44.1–79.6%) remained unused or only partially used at the time of data collection. Although this percentage decreased over time, it remained considerable even 9 years after cryopreservation. This finding supports the long-term storage of frozen sperm and highlights the need for continuous expansion of semen banks as the number of remaining frozen straws increases. This expansion requires not only increasing the number of storage containers but also enhancing administrative and logistical support for organizing international shipments and artificial inseminations, while maintaining robust safety protocols and high-quality service.

Overall, this study provides valuable insights into the use of frozen dog sperm by breeders in Belgium and the Netherlands. Although the findings may not be fully generalizable to other regions, the increasing popularity of sperm cryopreservation reflects the evolving landscape of canine reproductive management. Further studies should aim to quantify the proportion of breeders resorting to sperm cryopreservation and the extent of frozen sperm usage in breeding programs.

## Data Availability

The raw data supporting the conclusions of this article will be made available by the authors, without undue reservation.
