# Supplementary material for: Rising trends in the use of frozen dog sperm: a retrospective study in Belgium and the Netherlands
Source: Front Vet Sci. 2024 Nov 13;11:1499266. doi: 10.3389/fvets.2024.1499266 (PMC11598931; doi:10.3389/fvets.2024.1499266)
Supplement: Supplementary file 1 [file Table_1.DOCX]

Supplementary Material

# Supplementary tables

Supplementary Table 1. Distribution of dog breeds and corresponding number of individuals used for sperm cryopreservation.

| **Breed** | **Number of individuals** |
| --- | --- |
| Belgian Shepherd - Malinois | 215 |
| Golden Retriever | 59 |
| Labrador Retriever | 58 |
| German Shepherd | 51 |
| Dutch Shepherd | 42 |
| Border Collie | 41 |
| Bullmastiff | 39 |
| Belgian Shepherd - Tervueren | 38 |
| French Bulldog | 33 |
| Rottweiler | 31 |
| Rhodesian Ridgeback | 30 |
| Staffordshire Bull Terrier | 27 |
| Flat-coated Retriever | 25 |
| Stabijhoun | 23 |
| White Swiss Shepherd Dog | 23 |
| Bordeaux Dog | 21 |
| Briard | 21 |
| American Staffhordshire Terrier | 18 |
| Leonberger | 17 |
| Great Dane | 16 |
| Bouvier des Flandres | 15 |
| Bernese Mountain Dog | 13 |
| Bull Terrier | 13 |
| Chow Chow | 13 |
| Doberman | 13 |
| Groenendael | 13 |
| Lagotto Romagnolo | 13 |
| Cavalier King Charles Spaniel | 12 |
| Irish Soft Coated Wheaten Terrier | 12 |
| Newfoundland | 12 |
| Petit Basset Griffon Vendeen | 12 |
| Weimaraner | 12 |
| Finnish Lapphund | 11 |
| Shiba Inu | 11 |
| English Bulldog | 10 |
| Nova Scotia Tolling Retriever | 10 |
| Wetterhoun | 10 |
| Australian Shepherd | 9 |
| Bearded Collie | 9 |
| Landseer | 9 |
| Manchester Terrier | 9 |
| Afghan hound | 8 |
| Dachshund | 8 |
| German Shorthaired Pointer | 8 |
| Irish Setter | 8 |
| Irish Wolfhound | 8 |
| Shar-Pei | 8 |
| American Akita | 7 |
| Barbet | 7 |
| Boxer | 7 |
| Dalmatian | 7 |
| English Cocker Spaniel | 7 |
| Kooikerhondje | 7 |
| Beauceron | 6 |
| Mastiff | 6 |
| Poodle Standard | 6 |
| Spinone Italiano | 6 |
| Vizsla | 6 |
| Whippet | 6 |
| Alaskan Malamute | 5 |
| Beagle | 5 |
| Bloodhound (Saint-Hubert) | 5 |
| Bolognese | 5 |
| Cane Corso | 5 |
| Drentsche Patrijshond | 5 |
| German Wirehaired Pointer | 5 |
| Poodle Miniature | 5 |
| Samoyed | 5 |
| Shetland Sheepdog | 5 |
| Small Munsterlander | 5 |
| American Bulldog | 4 |
| American Cocker Spaniel | 4 |
| Australian Cattle dog | 4 |
| Basset Hound | 4 |
| Collie Rough | 4 |
| Giant Schnauzer | 4 |
| Gordon Setter | 4 |
| Old English Sheepdog | 4 |
| Parson Russel Terrier | 4 |
| Saarloos Wolfdog | 4 |
| Welsh Springer Spaniel | 4 |
| Akita Inu | 3 |
| Basset Artesien Normand | 3 |
| Borzoi | 3 |
| Chihuahua | 3 |
| Clumber Spaniel | 3 |
| Coton de Tulear | 3 |
| Grand Basset Griffon Vendeen | 3 |
| Greater Swiss Mountain Dog | 3 |
| Icelandic Shepdog | 3 |
| Irish Water Spaniel | 3 |
| Jack Russell Terrier | 3 |
| Lhasa Apso | 3 |
| Mastino Napoletano | 3 |
| Miniature Schnauzer | 3 |
| Pomeranian | 3 |
| Saluki | 3 |
| Scottish Deerhound | 3 |
| Siberian Husky | 3 |
| Sussex Spaniel | 3 |
| Thai Rhidgeback Dog | 3 |
| Welsh Corgi Cardigan | 3 |
| Australian Kelpie | 2 |
| Basenji | 2 |
| Black and Tan Coonhound | 2 |
| Border Terrier | 2 |
| Bracco Italiano | 2 |
| Broholmer | 2 |
| Cairn Terrier | 2 |
| Chinese Crested Dog | 2 |
| Cimarron Uruguayo | 2 |
| Continental Toy Spaniel | 2 |
| Curly-Coated Retriever | 2 |
| Dogo Argentino | 2 |
| English Springer Spaniel | 2 |
| Field Spaniel | 2 |
| Havanese | 2 |
| Hovawart | 2 |
| Japanese Chin | 2 |
| Laekenois (Belgian Sheherd) | 2 |
| Lancashire Heeler | 2 |
| Large Munsterlander | 2 |
| Little Lion Dog | 2 |
| Norwegian Buhund | 2 |
| Pharaoh Hound | 2 |
| Pyrenean Shepherd | 2 |
| Saint Bernard | 2 |
| Spanish Water Dog | 2 |
| Tibetan Mastiff | 2 |
| West Highland White Terrier | 2 |
| Affenpinscher | 1 |
| Australian Silky Terrier | 1 |
| Azawakh | 1 |
| Boston Terrier | 1 |
| English Pointer | 1 |
| English Setter | 1 |
| English Staffordshire Bull Terrier | 1 |
| Fila Brasileiro | 1 |
| Fox Terrier | 1 |
| French Spaniel | 1 |
| German Longhaired Pointer | 1 |
| German Pinscher | 1 |
| Greyhound | 1 |
| Griffon Bruxellois | 1 |
| Hokkaido | 1 |
| Irish Terrier | 1 |
| Kai | 1 |
| Keeshond | 1 |
| Pekingese | 1 |
| Perro dogo Mallorquin | 1 |
| Portuguese Water Dog | 1 |
| Pumi | 1 |
| Slovakian Wirehaired Pointer | 1 |
| Swedish Lapphund | 1 |
| Tatra Shepherd Dog | 1 |
| Tibetan Terrier | 1 |
| Welsh Terrier | 1 |

Supplementary Table 2. Frequencies and percentages of remaining frozen ejaculates from each year in the sperm banks.

| **Year** | **Number of cryopreserved ejaculates** | **Number of remaining ejaculates** | **Relative percentage of remaining ejaculates (%)** | **Cumulative number of remaining ejaculates** |
| --- | --- | --- | --- | --- |
| 2014 | 145 | 64 | 44.14 | 64 |
| 2015 | 193 | 90 | 46.63 | 154 |
| 2016 | 231 | 135 | 58.44 | 289 |
| 2017 | 283 | 156 | 55.12 | 445 |
| 2018 | 307 | 173 | 56.35 | 618 |
| 2019 | 351 | 181 | 51.57 | 799 |
| 2020 | 498 | 261 | 52.41 | 1060 |
| 2021 | 632 | 387 | 61.23 | 1447 |
| 2022 | 450 | 358 | 79.55 | 1805 |
